# Relationship between Serum Osteocalcin Levels and Non-Alcoholic Fatty Liver Disease in Adult Males, South China

**DOI:** 10.3390/ijms141019782

**Published:** 2013-09-30

**Authors:** Jun-Jie Liu, Yuan-Yuan Chen, Zeng-Nan Mo, Gui-Xiang Tian, Ai-Hua Tan, Yong Gao, Xiao-Bo Yang, Hai-Ying Zhang, Zhi-Xian Li

**Affiliations:** 1 Department of Ultrasound, First Affiliated Hospital of Guangxi Medical University, Nanning 530021, China; E-Mails: junjieliu008@163.com (J.-J.L.); YuanYuan_Chen01@163.com (Y.-Y.C.); GX_Tian1977@163.com (G.-X.T.); 2 Center for Personalized and Genomic Medicine, Guangxi Medical University, Nanning 530021, China; E-Mails: ZengNan_Mo@yeah.net (Z.-N.M.); YGao2011@126.com (Y.G.); 3 Institute of Urology and Nephrology, First Affiliated Hospital of Guangxi Medical University, Nanning 530021, China; 4 Center for Metabolic Disease and Diabetes, First Affiliated Hospital of Guangxi Medical University, Nanning 530021, China; E-Mail: AiHua_Tian@126.com; 5 Department of occupational health and environmental health, school of public health of Guangxi Medical University, Nanning 530021, China; E-Mails: Xiaobo_Yang93@yeah.net (X.-B.Y.); hy_zhang01@126.com (H.-Y.Z.)

**Keywords:** osteocalin, Insulin resistance, non-alcoholic fatty liver disease, ultrasonography

## Abstract

**AIM:**

To determine serum osteocalcin levels in South Chinese males with non-alcoholic fatty liver disease (NAFLD) and to examine the relation between serum osteocalcin and NAFLD.

**METHODS:**

Data were collected from 1683 men attending the Fangchenggang Area Male Healthy and Examination Survey (FAMHES) from September 2009 to December 2009. Serum osteocalcin was measured with electrochemiluminescence immunoassay. An abdominal ultrasonographic examination for all individuals was performed by two experienced ultrasonographers. The associations of serum osteocalcin with NAFLD were evaluated.

**RESULTS:**

The levels of serum osteocalcin were lower in 364 NAFLD participants than in 1319 non-NAFLD participants (24.51 ± 1.38 ng/mL *vs.* 20.81 ± 1.33 ng/mL, *p* < 0.001). Serum osteocalin level was associated with the scale of NAFLD (*r* = −0.150, *p* < 0.01). Serum osteocalin level tended to decrease with the scale of NAFLD. Binary logistic regression analysis showed that decreased ORs for NAFLD were observed from the first to the fourth osteocalcin quartiles.

**CONCLUSIONS:**

Our findings suggest that a lower serum osteocalcin level is associated with the presence of NAFLD.

## 1. Introduction

Non-alcoholic fatty liver disease (NAFLD) is a type of fatty liver disease when fat is deposited in the liver but not due to excessive alcohol use [1]. Some epidemiological surveys have showed that NAFLD has become a serious public health problem in China and even Asia [2–5]. To date, the mechanism of NAFLD is not fully understood. However, NAFLD is now considered to be a manifestation of metabolic syndrome (MetS) and it has been reported to relate to insulin resistance (IR) [6,7]. IR is almost a universal finding in NAFLD [8].

Osteocalcin, a hormone secreted by osteoblasts, can stimulate insulin expression in β-cells and protect animals from obesity and glucose intolerance [9]. Recent studies suggest that serum osteocalcin plays a key role in the pathogenesis of IR and energy expenditure [10,11]. Moreover, decreased serum osteocalcin levels have been reported in patients with metabolic syndrome [12]. Fernández-Real *et al.* thought that osteocalcin may play a role in the development of insulin resistance-associated fatty liver disease [13]. However, there are few studies that have explored the association between osteocalcin and NAFLD [14–16], which reported that in East Chinese men and Turkish patients, NAFLD patients were seen with a decreased serum osteocalcin level than in the controls. This prompted us to investigate the role of osteocalcin in the development of NAFLD.

Therefore, we conducted a cross-sectional study to investigate serum osteocalcin levels in South Chinese males with abdominal ultrasonography proven NAFLD, and to examine the relation of serum osteocalin and NAFLD.

## 2. Results and Discussion

Among 1683 individuals enrolled in this study, the prevalence of NAFLD was 21.74%. The characteristics of the participants were divided into non-NAFLD (*n* = 1319) and NAFLD (*n* = 364) groups shown in Table 1. Compared with participants in non-NAFLD group, those in NAFLD group had lower serum osteocalcin levels (*p* < 0.001) (Table 1). In non-NAFLD group, the mean of serum osteocalcin was 24.51 ng/mL with a SD of 1.38 ng/mL while in NAFLD group the mean of serum osteocalcin was 20.81 ng/mL with a SD of 1.33 ng/mL. All variables had statistical differences between these two groups (Table 1).

Age-adjusted Spearman correlation coefficient between osteocalcin and variables was shown in Table 2. There was a statistical negative correlation between osteocalcin and WC, BMI, TC, LDL-C, glucose, TG and ALT after adjustment for age (all *p* < 0.001). However, there was no statistically significant correlation between osteocalcin and HDL-C (*p* = 0.437). The strongest correlation was observed between osteocalcin and BMI (*r* = −0.246). The important finding in our study was that serum osteocalcin levels were statistically associated with the scale of NAFLD after adjustment of age and physical activity. Osteocalcin showed a decreased trend with the scale (*r* = −0.150, *p* < 0.001).

**Figure 1:**
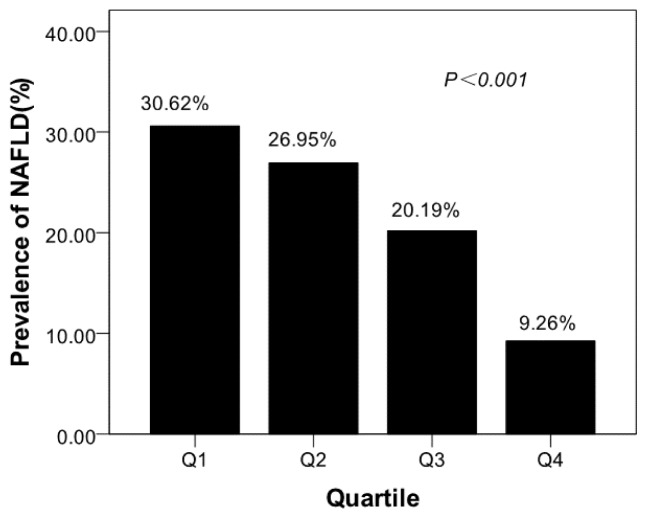
Prevalence of non-alcoholic fatty liver disease (NAFLD) in different quartiles of osteocalcin. The prevalence of NAFLD from the lowest to highest osteocalcin quartile were 30.62%, 26.95%, 20.19%, 9.26%, respectively (*p* < 0.001).

Participants with lower serum osteocalcin levels might be suffer from NAFLD. The prevalence of NAFLD of different quartiles was shown in Figure 1. The prevalence of NAFLD from the lowest to highest osteocalcin quartile were 30.62%, 26.95%, 20.19%, 9.26%, respectively (*p* < 0.001). The binary logistic regression analysis results were shown in Table 3. Decreased ORs for NAFLD were observed from the first to the fourth osteocalcin quartiles. Compared with the subjects in the fourth osteocalcin quartile, those in the first quartile had an OR of 4.32 (95% confidence interval (CI), 2.93–6.38); those in the second quartile had an OR of 3.54 (CI, 2.39–5.25) and those in the third quartile had an OR of 2.47 (CI, 1.65–3.71).

After the adjustment of age, BMI and physical activity, the ORs decreased obviously. Compared with the subjects in the fourth osteocalcin quartile, participants in the first quartile had an OR of 2.25 (CI, 1.42–3.55), those in the second quartile had an OR of 2.28 (CI, 1.44–3.63), and those in the third quartile had an OR of 1.83 (CI, 1.14–2.93).

In original studies on osteocalcin and insulin resistance, Lee *et al.* found that mice were lacking osteocalcin displayed decreased β-cell proliferation, glucose intolerance, and insulin resistance [9]. A great deal of interest followed this report and some studies tried to evaluate the relation between serum osteocalcin levels and metabolic syndrome [14,18–20], which showed that osteocalcin, plasma CK-18, and SREBF-1c polymorphism are associated with liver fibrosis and NAFLD. The recently reported association of osteocalcin with insulin resistant and metabolic syndrome prompts us to evaluate serum osteocalcin levels in patients with NAFLD.

Our investigation demonstrated that the level of serum osteocalcin in NAFLD group was lower than in non-NAFLD group (20.81 ± 1.33 ng/mL *vs.* 24.51 ± 1.38ng/mL; *p* < 0.001). One novel finding in our study was that serum osteocalcin levels were statistically associated with the scale of NAFLD after adjustment of age and physical activity. Osteocalcin showed a decreased trend with the scale of NAFLD (*r* = −0.150, *p* < 0.001). Moreover, after adjustment of age, physical activity and BMI, the binary logistic regression analysis results showed decreased ORs for NAFLD were observed from the first to the fourth osteocalcin quartiles. It is uncertain which mechanisms lead to lower osteocalcin levels in patients with NAFLD [21]. The relationship between serum osteocalcin levels and NAFLD could be hypothetically explained by the fact that osteocalcin increases insulin and adiponectin expression, decreases fat mass, improves insulin sensitivity and may thereby protect people against the development of NAFLD.

Some findings indicated that serum osteocalcin level was one of the predictors of insulin sensitivity and metabolic syndrome. [11,22–24] This study also kept consistent with prior reports. The glucose level in NAFLD group was higher than non-NAFLD group (5.57 ± 1.30 mmol/L *vs*. 5.20 ± 0.82 mmol/L; *p* < 0.001). Other variables in Table 1 had statistical differences between these two groups. We further found an inverse association between serum osteocalcin and fasting plasma glucose, BMI and waist circumference (Table 2).

Fatty liver disease is the most common cause of mild to moderate elevation of liver enzymes. Fernández-Real *et al.* found that circulating osteocalcin concentration was associated with parameters of liver fat infiltration (serum ALT and gamma glutamyl transferase levels) [13]. Consistent with their previous reports, we found serum osteocalcin levels to be inversely related to ALT. Compared with participants in non-NAFLD group, those with NAFLD had higher serum ALT levels (*p* < 0.001) (Table 1). In non-NAFLD group, the mean of serum osteocalcin was 36.90 U/L with a SD of 12.65 U/L while in NAFLD group the mean of serum ALT was 48.82 U/L with a SD of 15.25 U/L. In addition, serum ALT levels were linked to serum osteocalcin levels (*r* = −0.144, *p* < 0.001). The results implied that osteocalcin could be a potential novel marker to assess the progression of NAFLD.

There has been some research on the association of osteocalcin with NAFLD. To the best of our knowledge, there only one study has showed an association of decreased osteocalcin levels with the presence of NAFLD in a relatively larger population in East China [14]. However, two limitations in our study should be addressed. Because this study was performed in relatively healthy Chinese males, it had selection bias. However, such a large population-based sample limited the selection bias to some extent. It is well known that liver biopsy remains the best way to diagnose NAFLD and establish the presence of fibrosis, but it cannot be performed easily in a large population because of its invasiveness [12]. In fact, abdominal ultrasonography is more accessible than liver biopsy [25–27]. It is the optimal choice for NAFLD epidemiological surveys [28].

## 3. Experimental Section

### 3.1. Subjects

The Fangchenggang Area Male Health and Examination Survey was a population–based study conducted among non–institutionalized Chinese males aging from 17 to 88 years old in Guangxi. This study was designed to investigate the effects of environmental and genetic factors and their interaction with the development of age-related chronic diseases. A comprehensive demographic and health survey was conducted among 4303 men, who participated in a large-scale physical examination in the Medical Centre of Fangchenggang First People’s Hospital from September to December in 2009. Each method and potential risks were explained in detail to the participants, who gave written informed consent before the survey. In addition, the study received approval from ethics committee of Guangxi medical university.

Participants were included in this study based on the following criteria: (1) aged above 17 years old Fangchenggang area males; (2) having a stable and settled lifestyle.

Participants were excluded from this study based on the following criteria: (1) currently diagnosed with diabetes mellitus, coronary heart disease, stroke, hyperthyroidism, rheumatoid arthritis, and cancer; (2) currently diagnosed with hepatitis B virus (determined by the presence of hepatitis B surface antigen); with other hepatitis history and impaired hepatic function (alanine transaminase >2.0 times upper limit of normal); with liver cirrhosis or hepatic carcinoma; (3) taking any kind of medication (such as dehydrocortisone, methotrexate and so on) known to cause fatty liver’s images (images of brightechoes in liver parenchyma) during the previous year; (4) with excessive alcohol consumption (≥20 g/d, according to the published report [21]); (5) with incompleted follow-up clinical or laboratory examination, or ultrasound examination.

### 3.2. Epidemiological Survey

Data on demographic characteristics (age, education, occupation, *etc*.), lifestyle characteristics (alcohol consumption and physical activity), health status, and medical history were collected by using a standardized questionnaire (details were described by Tan *et al.* [12]). Body measurements including body weight and standing height were performed by a trained examiner using a standardized protocol. Body mass index (BMI) was calculated as weight (kilograms) divided by squared height (meters). The amount of alcohol intake was obtained from a series of questions including quantity of alcohol intake each time, times of alcohol intake each day, months of alcohol intake each year, years of alcohol intake, type and alcoholicity of beverage, drinking and dietary habits. From these data, we calculated the average daily and total alcohol assumption based on previous published method [3,29].

### 3.3. Ultrasonography

An abdominal ultrasonographic examination for all individuals was performed by two experienced ultrasonographer using a portable ultrasound device (GE, LOGIQ e, 5.0-MHz transducer, Fairfield, CT, USA). The liver of each participant was assessed for size, contour, echogenicity, structure and posterior beam attenuation.

The ultrasonographic criteria used to diagnose fatty liver disease included the presence of increased liver echogenicity (bright), stronger echoes in the hepatic parenchyma than in the renal parenchyma, vessel blurring, and narrowing of the lumen of the hepatic veins [23,29–31]. The degree of fatty liver disease on ultrasonography was divided into three scales (mild, moderate or severe) according to the criteria described by Saadeh *et al.* [23].

### 3.4. Laboratory Assessments

Overnight fasting venous blood specimens were drawn. Serum total cholesterol (TC), triglyceride (TG), high density lipoprotein cholesterol (HDL-C), low density lipoprotein cholesterol (LDL-C) and glucose levels were measured enzymatically on Dimension-RxL Chemistry Analyzer (Dade Behring, Newark, DE, USA) in the Department of Clinical Laboratory at the Fangchenggang First People’s Hospital. Serum osteocalcin was measured with electrochemiluminescence immunoassay on COBAS 6000 system E601 (Elecsys module) immunoassay analyzer (Roche Diagnostics, GmbH, Mannheim, Germany) with the same batch of reagents. The interassay coefficient of variation was 4.5%.

### 3.5. Quality Control

All researchers were given systematic training before the investigation. For further quality control, 5% of the questionnaires and blood sample results were randomly chosen for the re-examination; kappa analysis of these samples showed good consistency in the results of the diagnostic tests (data not shown). Abdominal ultrasonic examinations were performed by two experienced ultrasonographers using the same portable ultrasound device.

### 3.6. Statistical Analysis

Statistical analyses were performed using SPSS 18.0 software (IBM, Chicago, IL, USA). Descriptive results of continuous variables were expressed as either mean (±SD) or median and quartiles, and categorical data were expressed as percentage. The distribution of variables was analyzed with Kolmogorov-Smirnov test. Natural logarithmic transformations were performed so that osteocalcin values changed from a markedly skewed distribution to approximate normality. Basic characteristics of different groups were compared using Student’s *t* test or chi-square test when it is appropriate. The chi-square test was also used to compare the prevalence of NAFLD between different osteocalcin quartiles. Spearman correlation coefficients (adjusted for age) were calculated between serum osteocalcin and the following variables: waist circumference (WC), Glucose, BMI, ALT, TC, TG, HDL, and LDL. The association between serum osteocalcin levels and the scale of NAFLD was also evaluated. The binary logistic regression model was used to assess whether serum osteocalcin levels were associated with the presence of NAFLD. The odds ratio (OR) was calculated for the presence of NAFLD in quartiles of osteocalcin, with the participants in the highest quartile of osteocalcin considered as the reference group. Levels of statistical significance were set at *p* < 0.05.

## 4. Conclusions

Our findings suggest that lower serum osteocalcin levels are associated with measures of insulin resistance (fasting plasma glucose) and the presence of NAFLD. In the future, further studies are needed to establish the role of serum osteocalcin in patients with NAFLD.

## Figures and Tables

**Table 1:** Baseline characteristics of the subjects in non-NAFLD and non-alcoholic fatty liver disease (NAFLD) groups.

Characteristic	Non-NAFLD (*n* = 1319)	NAFLD (*n* = 364)	*p* value
Age (years)	36.69 ± 11.37	40.52 ± 10.18	<0.001
Osteocalcin (ng/mL)	24.51 ± 1.38	20.81 ± 1.33	<0.001
WC (cm)	77.74 ± 7.68	89.29 ± 6.84	<0.001
BMI (kg/m^2^)	22.25 ± 2.67	26.25 ± 2.74	<0.001
ALT (U/L)	36.90 ± 12.65	48.82 ± 15.25	<0.001
Glucose (mmol/L)	5.20 ± 0.82	5.57 ± 1.30	<0.001
TC (mmol/L)	5.60 ± 1.01	6.05 ± 1.06	<0.001
TG (mmol/L)	1.24 ± 0.92	2.44 ± 1.40	<0.001
HDL-C (mmol/L)	1.43 ± 0.29	1.30 ± 0.40	<0.001
LDL-C (mmol/L)	2.88 ± 0.79	3.24 ± 0.78	<0.001
Physical activity (*n*, %)			
Low *	848(64.3)	208(57.1)	
Moderate *	368(27.9)	131(36.0)	0.011
High *	103(7.8)	25(6.9)	

* Note: The physical activity level was classified as low, moderate, or high according to Guidelines for Data Processing and Analysis of the International Physical Activity Questionnaire (IPAQ) (available at: http://www.ipaq.ki.se/scoring.pdf). High: equivalent to approximately at least one hour per day or more, of at least moderate-intensity activity above the basal level of physical activity; Moderate: equivalent to half an hour of at least moderate-intensity physical activity on most days; Low: not meeting any of the criteria for either of the previous categories [17].

**Table 2:** Age-adjusted Spearman correlations between osteocalcin and variables.

Variable	Correlation coefficients	*p* value
WC	−0.223	<0.001
BMI	−0.246	<0.001
ALT	−0.151	<0.001
TC	−0.105	<0.001
HDL	0.019	0.437
LDL	−0.083	<0.001
Glucose	−0.094	<0.001
TG	−0.142	<0.001

**Table 3:** Binary logistic regression model examining associations of serum osteocalcin level and the presence of non-alcoholic fatty liver disease (NAFLD) Age-adjusted Spearman correlations between osteocalcin and variables.

Quartile of osteocalcin (ng/mL)	Odds ratio (95%CI)	*p* value	Adjusted ^1^ Odds ratio (95%CI)	*p* value	Adjusted ^2^ Odds ratio (95%CI)	*p* value
Q4 (>29.21)	1		1		1	
Q3 (23.41–29.21)	2.47 (1.65–3.71)	<0.001	2.30 (1.53–3.46)	<0.001	1.83 (1.14–2.93)	=0.012
Q2 (18.98–23.41)	3.54 (2.39–5.25)	<0.001	3.10 (2.08–4.63)	<0.001	2.28 (1.44–3.63)	<0.001
Q1 (<18.98)	4.32 (2.93–6.38)	<0.001	3.60 (2.41–5.39)	<0.001	2.25 (1.42–3.55)	<0.001
